# A step in the right direction: the evidence for treating of tinea pedis and onychomycosis in people with diabetes

**DOI:** 10.1186/1757-1146-4-S1-P34

**Published:** 2011-05-20

**Authors:** Lisa Matricciani, Kerwin Talbot, Sara Jones

**Affiliations:** 1 University of South Australia, Adelaide, SA, 5000, Australia

## Background

Effective treatment of tinea pedis and onychomycosis is crucial for patients with diabetes as these infections may lead to foot ulcers and eventual lower limb amputation. Although numerous studies have assessed the effectiveness of antifungal drug and treatment regimens, most exclude patients with diabetes and examine otherwise healthy individuals. While these studies are useful, results cannot necessarily be extrapolated to patients with diabetes. The purpose of this study was to therefore identify the best evidence-based treatment options for tinea pedis or onychomycosis in people with diabetes.

## Methods

The question for this systemic review was what evidence is there for antifungal treatment interventions for adults with tinea pedis and/or onychomycosis in people with diabetes. A systematic literature search of four electronic databases (Scopus, EbscoHost, Ovid, Web of Science) was undertaken using the search terms ‘‘onychomycosis” or “tinea pedis” or “athlete’s foot” or “tinea unguium” and ‘‘diab*’’ and “treat*” (15/10/10). No language or date restrictions were set. Studies that examined the efficacy and/or safety of treatment options for people with diabetes were included in this study.

## Results

Preliminary results of this study indicate a scarcity of studies specifically investigating antifungal treatments of tinea pedis and onychomycosis in patients with diabetes. Of the seven studies identified to date, all examined the efficacy and/or safety of antifungal agents for the treatment of onychomycosis. Of the antifungal agents assessed, topical ciclopirox nail lacquer, oral terbinafine and oral itraconazole were found both safe and effective for treatment. To date, no studies investigating the treatment for tinea pedis have been identified. Although suggestions that combination drug therapy and physical debridement are effective treatment strategies, studies to confirm these suggestions have never been conducted.

## Conclusions

The results of this study indicate further research is needed to determine the best evidence-based treatment options for tinea pedis and onychomycosis in patients with diabetes. Areas for future research include studies that investigate the treatment for tinea pedis, the effectiveness of other antifungal drugs and dosing regimens, combination drug therapy and physical debridement. Such research may identify more effective treatment options that may reduce the incidence of diabetic foot ulcers and associated complications.

